# Predicting preference-based utility values using partial proportional odds models

**DOI:** 10.1186/1756-0500-7-438

**Published:** 2014-07-08

**Authors:** Roberta Ara, Ben Kearns, Ben A vanHout, John E Brazier

**Affiliations:** 1 University of Sheffield, School of Health and Related Research, 30 Regent Street, Regent Court, Sheffield S1 4DA, UK

**Keywords:** EQ-5D, Mapping, Regression, Partial proportional odds, Ordered logit, Response mapping

## Abstract

**Background:**

The majority of analyses on utility data have used ordinary least square (OLS) regressions to explore potential relationships. The aim of this paper is to explore the benefits of response mapping onto health dimension profiles to generate preference-based utility scores using partial proportional odds models (PPOM).

**Methods:**

Models are estimated using EQ-5D data collected in the Health Survey for England and the predicted utility scores are compared with those obtained using OLS regressions. Explanatory variables include age, acute illness, educational level, general health, deprivation and survey year. The expected EQ-5D scores for the PPOMs are obtained by weighting the predicted probabilities of scoring one, two or three for the five health dimensions by the corresponding preference-weights.

**Results:**

The EQ-5D scores obtained using the probabilities from the PPOMs characterise the actual distribution of EQ-5D preference-based utility scores more accurately than those obtained from the linear model. The mean absolute and mean squared errors in the individual predicted values are also reduced for the PPOM models.

**Conclusions:**

The PPOM models characterise the underlying distributions of the EQ-5D data better than models obtained using OLS regressions. Additional research exploring the effect of modelling conditional responses and two part models could potentially improve the results further.

## Background

Many health care policy decision-making bodies require that economic evidence used to support submissions are reported in terms of the cost per quality adjusted life years (QALY) [1-5]. The QALY is a metric which combines both life expectancy and health related quality of life (HRQoL) where the HRQoL is informed by a preference-based measure of health such as the EQ-5D [6,7]. As a consequence of submission requirements, there has been a substantial growth in the number of articles describing the results of statistical regression models involving preference-based measures. This research may be performed either because the preferred preference-based measure is not available in a particular clinical dataset, or the cost-effectiveness model structure requires a method to predict changes in HRQoL values based on changes in clinical variables over time.

The majority of publications describing analyses of these types of data tend to describe regressions estimated using ordinary least squares (OLS) [8]. Although the statistical models obtained using OLS perform well on the aggregate level, they tend to under-predict at the top and over-predict at the bottom of the index and can predict values outside the actual index range [9,10]. HRQoL data are generally multi-modal with a mass at full health (i.e. EQ-5D = 1) and simple linear models are not appropriate [11]. Some researchers have explored alternatives such as generalised linear models with random effects, adjusted least square regression models, weighted least squares, Tobit models, and censored least absolute deviation models [8]. While these appear to provide little or no improvements in terms of predictive abilities or fit, an adjusted censored mixture model has been shown to improve predictive abilities compared to a model obtained using OLS in rheumatoid arthritis [11].

An alternative approach to mapping directly onto the preference-based index is to predict the original responses to the health dimension questions (‘response mapping’). Studies reporting these types of analyses have typically used multinomial logistic regressions and to date the benefits have appeared to be minimal [12,13]. Multinomial logistic models are generally used when the dependent variable is nominal (i.e. the categories cannot be ordered in any meaningful way). As the responses for the health dimensions can be ranked according to the level of problems (i.e. none, some or extreme) an ordered logistic model (OLM) which ranks the responses for the health dimensions may be more appropriate. The aim of this paper is to develop the response mapping approach by exploring the use of partial proportional odds models (PPOM). These models are less restrictive than the parallel lines fit in the OLM but retain the ordered nature of the responses for the dependent variables [14]. The results obtained are compared to those obtained using OLS regressions as these are the most widely used in this area.

## Methods

### EQ-5D

The EQ-5D is a self-administered questionnaire which covers five health dimensions: mobility, self-care, usual activities, pain/discomfort, anxiety/depression. Each dimension in the descriptive system has three levels (no problems, some problems, extreme problems), producing a maximum of 243 (3^5) possible health states. Full health, with no impairment on all five dimensions, is defined by 11111. The worst possible health state, with maximum impairment on all five dimensions, is defined by 33333. EQ-5D preference-based utility values for each of the 243 health states are derived using the utility weights obtained from a sample of the British general population using time-trade-off methods [6]. The resulting EQ-5D preference-based index ranges from 1 (health state 11111) to-0.594 (health state 33333).

### Data

The Health Survey for England (HSE) is an annual cross-sectional survey of randomly selected residents of private households in England. Ethical approval for the HSE was obtained from the London Multi-Centre Research Ethics Committee and the data are freely available to download for academic purposes. Five cycles (years 2003, 2004, 2005, 2006 and 2008) of the HSE collected EQ-5D data, a generic quality of life instrument, and these datasets were combined for the purpose of this study [15-19]. Analyses were conducted using sub-groups of respondents who had completed the full EQ-5D questionnaire and indicated they had one of the following prevalent self-reported limiting long term illnesses (LLTI): cardiovascular disease (CVD), diabetes, mental health conditions, musculoskeletal conditions, nervous system disorders, respiratory problems.

Based on their known effects on HRQoL, and at the request of the commissioners, the following variables were selected as explanatory variables in the regressions: age, age^2^, education level, acute sickness within the previous two weeks, general health (self-reported by patients), and deprivation (based on the Overall Index of Multiple Deprivation) [20]. Education level was categorised as no formal qualification (base), GCSE O level or NVQ2/3 (GCSE), full time student or higher education below degree level including A level (A level), NVQ4/5 or degree (degree). Acute sickness was categorised as 0 days (base), 1 to 6 days (Sick 1), 6 to 13 days (Sick 2), and the maximum recall period of 14 days (Sick 3). General health was categorised as very good (GHVG), good (GHG), fair (base), bad (GHB), or very bad (GHVB), while deprivation was categorised as: least (base), a little deprived, very deprived, or most deprived (see Additional file 1).

### Statistical models obtained for each of the LLTIs

A simple linear model was generated using an OLS regression. The dependent variable was the EQ-5D preference-based utility, with age, age^2^, acute illness, education level, general health, deprivation, and survey year included as explanatory variables:

$$\mathrm{EQ}-5D\;=\alpha +\beta_{1}x_{1}+\beta_{2}x_{2}+\;\ldots \;\ldots \;\ldots \;+\beta_{n}x_{n}+\epsilon$$

EQ-5D represents the EQ-5D preference-based index, α represents the constant, the β’s represent the weights given to the various explanatory variables, and ϵ represents the error term. As has been discussed in the literature, linear models such as these do not deal with the non normal characteristics typically observed in EQ-5D data such as a mass at full health (EQ-5D = 1), a multimodal distribution, a long negative skew and the bounds of the index [11]. Predictions from these models are by definition concentrated around the mean (which is generally in the upper 50% of the index), consequently the models under-predict values at the top of the index and over-predict values at the bottom of the index [9]. This will be problematic when predicting mean values outside the inter quartile range or when predicting changes in mean values over time. In addition, when mean values are relatively high or low, the predicted values can exceed the limits of the index.

William’s generalised ordered logit/partial proportional odds models (referred to as the PPOM) with three dependent variables (the probability of scoring none, some or extreme problems) were obtained [14] for each of the five EQ-5D health dimensions:

$$\begin{array}{l} p\;\left(d_{i}=1\right)=\frac{\exp\;\left(\beta_{1}^{i}x_{1}+\beta_{2}^{i}x_{2}+....\beta_{n}^{i}x_{n}-k_{1}^{i}\right)}{1-\exp\;\left(\beta_{1}^{i}x_{1}+\beta_{2}^{i}x_{2}+....\beta_{n}^{i}x_{n}-k_{1}^{i}\right)}\\ p\;\left(d_{i}=2\right)=\frac{\exp\;\left(\beta_{1}^{i}x_{1}+\beta_{2}^{i}x_{2}+....\beta_{n}^{i}x_{n}-k_{2}^{i}\right)}{1-\exp\;\left(\beta_{1}^{i}x_{1}+\beta_{2}^{i}x_{2}+....\beta_{n}^{i}x_{n}-k_{2}^{i}\right)}-p\;(d_{i}=1)\\ p\;\left(d_{i}=3\right)=1-p\;(d_{i}=1)-p(d_{i}=2) \end{array}$$

Here the β’s reflect the weight given to the various independent variables and the *k*’s define the separation between the probabilities (or cut points). In the case of the PPOM, the βs may differ across values of *d* (i.e. the regression lines may not be parallel). An advantage of using PPOMs is that it is more parsimonious than the generalised ordered logit model, where all the βs differ, but unlike the multinomial logit model, the order of the responses is retained.

EQ-5D predictions for the OLS models were obtained in the normal manner using the OLS βs. As the logit models predict a range of probabilities on defined outcomes rather than a single value point, EQ-5D predictions were calculated by estimating an expectation using the EQ-5D scores for each of the 243 possible health states weighted by the probabilities of being in these health states. For example, if the models gave probabilities of 0.70, 0.2 and 0.1 for scoring level 1, 2 or 3 respectively for each of the five health dimensions, the preference based weights for each of dimensions levels were adjusted by the corresponding probability and the results summed to give the expected value. As this method produces the average expected score for each individual, as opposed to one of the 243 possible EQ-5D scores, the predicted values were not expected to replicate the actual EQ-5D scores exactly. For example, using the expected scores, it is not possible to generate a score of one.

Goodness of fit was assessed using standard summary statistics, and the ability of the statistical models to predict EQ-5D scores was assessed using the mean absolute errors (MAE), and root mean squared errors (RMSE). Errors in predictions were compared for sub-groups across the EQ-5D range (EQ-5D < 0; 0 ≤ EQ-5D < 0.5; 0.5 ≤ EQ-5D < 0.75; EQ-5D ≥ 0.75). The ability to represent the characteristics of the data was assessed graphically.

## Results

Comparing the LLTIs, there are substantial differences in the proportions of respondents who have problems in each of the five health dimensions, reflecting the different aspects of health affected by the particular condition (Table 1). For example 47% of respondents with respiratory conditions have problems with pain/discomfort compared to 80% of respondents with musculoskeletal conditions. Similarly, approximately 80% of respondents with mental health conditions have problems with anxiety/depression compared to 27% of respondents with CVD.

**Table 1:** Proportion of respondents indicating problems on the five health dimensions

	**CVD**	**Diabetes**	**Mental health conditions**	**Musculoskeletal conditions**	**Nervous system**	**Respiratory problems**
n	7,998	4,513	1,901	11,290	2,236	5,110
EQ-5D (mean)	0.7242	0.7417	0.5980	0.6361	0.6483	0.7646
Full health (%)	31.5	35.4	13.1	15.1	22.9	41.5
Mobility (%)						
No problem	55.4	61.6	65.2	45.4	52.6	67.0
Some problem	44.3	38.0	34.7	54.3	46.8	32.9
Extreme problem	0.3	0.4	0.1	0.3	0.6	0.2
Self Care (%)						
No problem	85.8	87.5	84.2	82.3	80.0	88.9
Some problem	13.3	11.5	15.3	16.8	18.2	10.5
Extreme problem	0.9	1.0	0.5	0.9	1.8	0.6
Usual activities (%)						
No problem	62.5	66.9	55.4	52.4	52.3	70.0
Some problem	32.0	28.1	40.0	41.8	39.6	25.6
Extreme problem	5.6	5.0	4.6	5.8	8.1	4.4
Pain/discomfort (%)						
No problem	41.8	45.2	46.9	20.0	33.8	53.1
Some problem	49.0	45.6	42.9	64.7	51.7	38.5
Extreme problem	9.3	9.2	10.2	15.4	14.5	8.4
Anxiety/Depression (%)						
No problem	73.4	73.8	21.6	71.3	64.8	73.0
Some problem	23.8	23.2	53.6	25.3	29.6	23.4
Extreme problem	2.8	3.0	24.8	3.4	5.6	3.6

**Figure 1:**
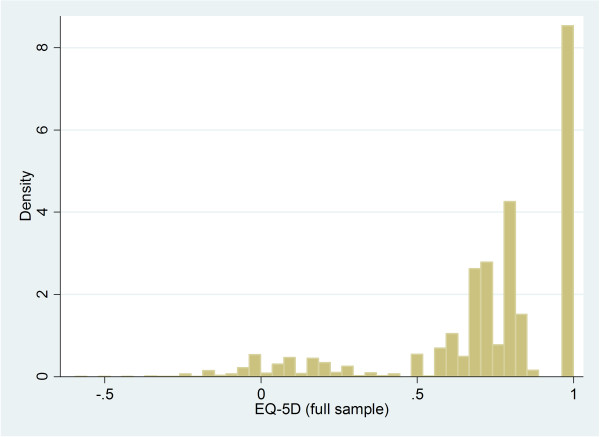
Distribution of EQ-5D scores for the full sample.

The individual EQ-5D scores cover the full range (-0.594 to 1) and there is little variation in the mean scores for the sub-groups across the survey year (Additional file 1). The largest variation is observed in the sub-group with musculoskeletal conditions (range 0.610 in 2008 to 0.652 in 2003) whilst the smallest variation is observed in the sub-group with CVD (range 0.716 in 2006 to 0.740 in 2003). The EQ-5D scores are not normally distributed irrespective of survey year or health condition and exhibit a long negative skew, a mass at full health, a second group centred around approximately 0.75 and a third group centred around approximately 0.2 (Figure 1). The proportion of respondents scoring full health (Table 1) is greatest in the sub-group with respiratory conditions (approximately 40%) and smallest in the sub-group with mental health conditions (approximately 13%).

### Statistical models

The result for the musculoskeletal condition is used as an exemplar (Table 2) as it has the largest sample and additional results are available online (Additional file 2). With the exception of the survey years, the majority of coefficients in the OLS models (Table 2 and Additional file 2) are statistically significant (p < 0.05) and all have the expected sign. For example, the coefficients for acute illness (Sick 1, Sick 2 and Sick 3) are negative and significant and the coefficients for deprivation are positive and negative for the least deprived, and most deprived, reflecting the increase and decrease in EQ-5D score respectively relative to the baseline (least deprived). The coefficients for the explanatory variables in the logit models are not as straightforward to interpret but if the relationship between the explanatory variable and HRQoL is negative, one would expect the corresponding coefficient in the logit model to be positive (increasing the probability of scoring 2 or 3 on the health dimension indicating a decrease in HRQoL).

**Table 2:** Model coefficients for the LLTI musculoskeletal conditions

**Method**	**OLS**		**PPOM**^**a**^		**PPOM**		**PPOM**		**PPOM**		**PPOM**	
**DV**	**EQ-5D**		**MOBILITY**		**SELF CARE**		**USUAL ACTIVITIES**		**PAIN**		**ANXIETY/ DEPRESSION**	
	**Beta**	**SE**	**Beta**	**SE**	**Beta**	**SE**	**Beta**	**SE**	**Beta**	**SE**	**Beta**	**SE**
**n**	**11290**		**11290**		**11290**		**11290**		**11290**		**11290**	
Age	-0.0006	0.00	-0.0174	0.01	-0.0159	0.01	-0.0263	0.01**	0.0727	0.01***	0.0007	0.01
			-0.0159	0.01	-0.0159	0.01	-0.0756	0.02***	0.0358	0.01**	-0.0178	0.01*
Age^2^	0.0000	0.00	0.0005	0.00***	0.0002	0.00*	0.0002	0.00**	-0.0007	0.00***	-0.0001	0.00*
			0.0004	0.00***	0.0004	0.00***	0.0008	0.00***	-0.0004	0.00**	-0.0001	0.00*
Sex	-0.0268	0.01***	-0.0001	0.05	-0.0783	0.07	0.0447	0.06	0.3204	0.04***	0.3648	0.05***
			-0.0783	0.07	-0.0783	0.07	-0.4889	0.10***	0.3204	0.04***	0.3648	0.05***
GCSE	0.0644	0.01***	-0.2323	0.10*	-0.3219	0.14*	0.1639	0.09	-0.5591	0.08***	-0.0961	0.09
			-0.3219	0.14*	-0.3219	0.14*	0.1639	0.09	-0.5591	0.08***	-0.9563	0.28**
A level	0.0271	0.01***	0.0136	0.08	-0.2329	0.10*	0.1734	0.08*	-0.1541	0.06*	-0.0820	0.07
			-0.2329	0.10*	-0.2329	0.10*	-0.2122	0.15	-0.1541	0.06*	-0.6695	0.18***
Degree	0.0196	0.01**	-0.1360	0.07*	0.0578	0.08	0.1871	0.07**	-0.0639	0.06	-0.1506	0.06*
			0.0578	0.08	0.0578	0.08	-0.1475	0.12	-0.0639	0.06	-0.5476	0.14***
Sick 1	-0.0641	0.01***	0.0109	0.12	0.2605	0.13*	0.6591	0.12***	0.5541	0.17**	0.2273	0.10*
			0.2605	0.13*	0.2605	0.13*	0.0714	0.21	0.0445	0.13	0.2273	0.10*
Sick 2	-0.0827	0.01***	0.1171	0.13	0.2757	0.11*	0.5660	0.12***	0.2097	0.10*	0.3250	0.10***
			0.2757	0.11*	0.2757	0.11*	0.0606	0.18	0.2097	0.10*	0.3250	0.10***
Sick 3	-0.1318	0.01***	0.1883	0.08*	0.2513	0.07**	1.0277	0.08***	0.4156	0.07***	0.1320	0.06*
			0.2513	0.07**	0.2513	0.07**	0.5188	0.12***	0.4156	0.07***	0.1320	0.06*
A little deprived	0.0142	0.01*	0.0158	0.07	-0.0453	0.10	-0.0225	0.07	-0.1409	0.06*	-0.0639	0.07
			-0.0453	0.09	-0.0453	0.10	-0.0225	0.07	-0.1409	0.06*	-0.0639	0.07
Very deprived	-0.0243	0.01***	0.0902	0.07	-0.0183	0.08	0.1438	0.06*	0.1138	0.06	0.0521	0.06
			-0.0183	0.08	-0.0183	0.08	0.1438	0.06*	0.1138	0.06	0.0521	0.06
Most deprived	-0.0402	0.01***	0.2260	0.08**	0.1713	0.08*	0.0053	0.07	0.0890	0.06	0.0534	0.06
			0.1713	0.08*	0.1713	0.08*	0.0053	0.07	0.0890	0.06	0.4140	0.12***
GHVG	0.1589	0.01***	-0.6702	0.09***	-0.2509	0.17	-0.9378	0.10***	-0.8494	0.08***	-1.1910	0.11***
			-0.2509	0.17	-0.2509	0.17	-0.0784	0.28	-0.8494	0.08***	-1.1910	0.11***
GHG	0.1162	0.01***	-0.5119	0.06***	-0.3853	0.10***	-0.6314	0.06***	-0.4466	0.06***	-0.5854	0.06***
			-0.3853	0.10***	-0.3853	0.10***	-0.2158	0.16	-0.4466	0.06***	-0.5854	0.06***
GHB	-0.2528	0.01***	0.5150	0.10***	0.6371	0.08***	0.4704	0.08***	0.1015	0.16	0.3935	0.07***
			0.6371	0.08***	0.6371	0.08**	0.4704	0.08***	0.9030	0.08***	1.0075	0.13***
GHVB	-0.4133	0.02***	0.7894	0.21***	0.7815	0.12***	0.3456	0.20	1.2408	0.11***	0.6730	0.11***
			0.7815	0.12***	0.7815	0.12***	1.1437	0.13***	1.2408	0.11***	1.5748	0.15***
Year 2004	-0.0029	0.01	-0.0830	0.09	0.0390	0.12	-0.1017	0.09	0.0786	0.08	0.0680	0.08
			0.0390	0.11	0.0390	0.12	-0.1017	0.09	0.0786	0.08	0.0680	0.08
Year 2005	0.0102	0.01	-0.0803	0.08	0.0903	0.10	-0.1103	0.07	0.0625	0.07	-0.1521	0.07*
			0.0903	0.10	0.0903	0.10	-0.1103	0.07	0.0625	0.07	-0.1521	0.07*
Year 2006	-0.0079	0.01	0.0729	0.08	0.1224	0.09	-0.0250	0.07	0.0394	0.06	-0.0936	0.07
			0.1224	0.09	0.1224	0.09	-0.0250	0.07	0.0394	0.06	-0.0936	0.07
Year 2008	-0.0229	0.01**	0.0690	0.08	0.0706	0.09	-0.0570	0.07	0.2080	0.06**	0.0682	0.06
			0.0706	0.09	0.0706	0.09	-0.0570	0.07	0.2080	0.06**	0.0682	0.06
Mobility					1.5026	0.12***	1.8501	0.06***	1.6166	0.08***	0.0759	0.06
					1.5026	0.12***	1.8501	0.06***	1.2556	0.11***	0.0759	0.06
Self care			1.0975	0.12***			2.2145	0.13***	-0.3805	0.14**	0.4749	0.06***
			1.5026	0.12***			1.4599	0.09***	0.5833	0.07***	0.4749	0.06***
Usual act			1.7782	0.06***	1.6862	0.08***			1.2432	0.09***	0.2970	0.05***
			2.4137	0.22***	2.4137	0.22***			0.9100	0.07***	0.2970	0.05***
Pain			1.4225	0.07***	0.6329	0.07***	1.1054	0.07***			0.4400	0.05***
			-0.2547	0.19	-0.2547	0.19	0.8089	0.10***			0.4400	0.05***
Anxiety			0.0547	0.06	0.4228	0.05***	0.2744	0.05***	0.4001	0.05***		
			0.4228	0.05***	0.4228	0.05***	0.2744	0.05***	0.4001	0.05***		
Constant	0.7578	0.02***	-7.0445	0.36***	-9.3774	0.50***	-7.4743	0.35***	-4.5057	0.32***	-2.8284	0.28***
			-14.063	0.89***	-14.062	0.89***	-8.6473	0.71***	-8.4218	0.45***	-4.7918	0.37***

^a^As the PPOMs comprise of two equations, each explanatory variable has two rows with the two Beta coefficients (and corresponding SE). *p < 0.05; **p < 0.01; ***p < 0.001.

The sign and statistical significance of the coefficients in the PPOMs (Table 2, see Additional file 2) are both health dimension and condition specific, reflecting the differences in the relationships with the particular health dimensions for each of the health conditions. The coefficients in the PPOMs also demonstrate the relationships can vary substantially for the different levels within a health dimension, and in some cases can change direction. For example, looking at the mobility dimension coefficients for the survey years 2004 and 2008 for respiratory conditions. The negative coefficients for the first equation (contrasting no problem to some problems and extreme problems) indicate an increase in the likelihood of being in the current or lower category. Conversely, the positive coefficients for the second equation (contrasting no problem and some problem with extreme problems), indicate an increase in the likelihood of being in a higher category than the current one.

### Errors in predicted values

While the OLS models are the most accurate in predicting the mean EQ-5D score for each of the LLTIs and surveys (Table 3), when comparing the dispersion of the actual and predicted EQ-5D scores, the OLS predictions cover a much smaller range, do not predict negative scores and predict values greater than one. The errors in the values predicted using the PPOMs are substantially smaller than those predicted using the OLS irrespective of the LLTI. For the OLS predictions, MAEs (RMSEs) range from 0.149 to 0.212 (0.208 to 0.269) compared to 0.110 to 0.169 (0.142 to 0.206) for the PPOM predictions (Table 3). When sub-grouping by actual EQ-5D score, the PPOM out-perform the OLS in terms of both MAEs and RMSEs, with the RMSEs between 10% and 50% (for EQ-5D scores smaller than 0) smaller for the PPOM predictions (Table 4).

**Table 3:** Actual and predicted EQ-5D scores

	**n**	**EQ-5D**	**OLS**	**PPOM**	**OLS**	**PPOM**	**OLS**	**PPOM**
		**mean (min,max)**	**mean (min,max)**	**mean (min,max)**	**Mean absolute error**		**Root mean squared error**	
CVD	7,998	0.724	0.724	0.695	0.166	0.115	0.226	0.150
		(-0.594,1.00)	(0.096, 1.04)	(-0.195, 0.896)				
Diabetes	4,460	0.739	0.739	0.707	0.163	0.115	0.222	0.147
		(-0.594,1.00)	(0.068,1.06)	(-0.231,0.903)				
Mental health	1,901	0.598	0.598	0.584	0.205	0.16	0.26	0.199
		(-0.484,1.00)	(0.033,0.962)	(-0.138,0.876)				
Musculoskeletal	11,290	0.636	0.636	0.621	0.174	0.12	0.236	0.165
		(-0.594,1.00)	(0.013,0.955)	(-0.263,0.885)				
Nervous system	2,236	0.648	0.648	0.626	0.186	0.131	0.246	0.170
		(-0.594,1.00)	(-0.016,1.01)	(-0.273,0.894)				
Respiratory	4,943	0.757	0.758	0.723	0.156	0.119	0.216	0.150
		(-0.484,1.00)	(0.128,1.02)	(-0.306,0.904)				

**Table 4:** Errors in predicted scores sub-grouped by EQ-5D index

			**Actual**	**Predicted**		**RMSE in predicted scores**	
Sub-grouped by actual EQ-5D scores		n	EQ-5D	OLS	PPOM	OLS	PPOM
EQ-5D < 0	CVD	370	-0.085	0.422	0.160	0.540	0.278
	Diabetes	203	-0.104	0.385	0.109	0.531	0.253
	Mental Health disorders	137	-0.124	0.300	0.125	0.457	0.276
	Musculoskeletal	775	-0.081	0.340	0.137	0.461	0.253
	Nervous System	169	-0.113	0.290	0.106	0.441	0.251
	Respiratory	218	-0.092	0.401	0.153	0.523	0.273
0 ≤ EQ-5D < 0.5	CVD	723	0.179	0.531	0.414	0.409	0.299
	Diabetes	375	0.169	0.520	0.400	0.411	0.301
	Mental Health disorders	450	0.241	0.487	0.453	0.310	0.264
	Musculoskeletal	1399	0.153	0.474	0.399	0.381	0.304
	Nervous System	302	0.176	0.475	0.383	0.374	0.284
	Respiratory	428	0.181	0.511	0.408	0.392	0.293
0.5 ≤ EQ-5D < 0.75	CVD	2657	0.667	0.660	0.615	0.165	0.109
	Diabetes	1315	0.668	0.666	0.616	0.174	0.117
	Mental Health disorders	535	0.663	0.594	0.555	0.185	0.170
	Musculoskeletal	4600	0.670	0.620	0.597	0.165	0.123
	Nervous System	728	0.660	0.614	0.583	0.176	0.135
	Respiratory	1209	0.666	0.651	0.609	0.179	0.125
EQ-5D ≥ 0.75	CVD	4248	0.923	0.823	0.840	0.158	0.114
	Diabetes	2567	0.926	0.837	0.846	0.153	0.111
	Mental Health disorders	779	0.887	0.717	0.761	0.221	0.151
	Musculoskeletal	4516	0.874	0.753	0.797	0.175	0.114
	Nervous System	1037	0.902	0.781	0.812	0.190	0.124
	Respiratory	3088	0.933	0.859	0.851	0.146	0.112

## Discussion and conclusions

In this article we compare the results obtained when predicting EQ-5D scores directly using the results of OLS regressions, to EQ-5D scores obtained indirectly using response mapping and a methodology which retains the ordered nature of the original responses for the health dimensions. We found that while the OLS and PPOM models produce mean utility values that closely approximate the actual mean values, the OLS predict distributions that have little resemblance to the actual distributions. In direct comparison, the distributional characteristics (mass at full health, multi-model distribution, long negative skew) of the EQ-5D data are captured and described better in the predictions from the PPOM models. The EQ-5D scores estimated using the logit predictions are constrained by the EQ-5D index while the OLS predictions are not and predict values greater than one. The RMSEs reflect these differences with substantially larger errors in the OLS predictions. This could be particularly important when using the models to estimate differences between sub-groups in economic models, or when adjusting for case-mix using individual predictions to compare differences between providers or changes over time.

When predicting scores for sub-groups across the EQ-5D range, the logit models out-perform the OLS across all the LLTIs and compare favourably with results in the literature. For a sub-group with actual EQ-5D scores below 0, when mapping between the SF-12 and the EQ-5D, Gray *et al*. report a mean squared error (MSE) and MAE of 0.166 and 0.304 respectively for response mapping using multinomial logit regressions (compared to 0.174 and 0.373 respectively for direct mapping using an OLS regression) [12]. Using the predictions for respondents with CVD as an exemplar, the MSE and MAE in our study were 0.077 and 0.245 respectively for response mapping using PPOMs (compared to 0.292 and 0.507 respectively for direct mapping using OLS regression). We chose to generate the expected values to enable comparison with the expected scores predicted from the OLS as opposed to simulating a sample of EQ-5D scores which could explain the differences in results.

There are limitations with the dataset used in this study. For example, the HSE sampling mechanism excludes inhabitants of hospitals, residential, and nursing homes, hence the actual mean EQ-5D scores for the LLTIs may be slightly lower than those reported in this study. In addition, the LLTIs are broadly defined and self-reported as opposed to clinically diagnosed by a doctor. This may introduce an element of bias as respondents may indicate they have a condition which has not been medically diagnosed. Conversely, respondents may indicate they do not have a particular condition which has actually been medically diagnosed. As the objective of the study was to compare the predicted values obtained using the different methodologies, as opposed to examining the mean EQ-5D scores for particular conditions, the limitations with the data should not affect the findings of the analyses. However, the mean values for the particular sub-groups may not be representative of the actual values.

The PPOM response mapping appears promising and there are several areas where additional research is warranted. A substantial proportion (range: 13% to 42%) of the respondents in each of the LLTI sub-groups indicated they were at full health (EQ-5D = 1) which suggests that a two-part model may be appropriate. A second area that could be developed is the order in which the EQ-5D questionnaire is completed (i.e. mobility followed by self care, usual activities, pain/discomfort, anxiety/depression). Intuitively one would expect that if someone scores no problem on the first four of the health dimensions, they are more likely to score no problems on the last dimension. Conversely, if they score extreme problems on the first four dimensions, it is unlikely that they will score no problems on the fifth dimension. We generated PPOMs for each health dimension independently, using the responses to the other four dimensions as explanatory variables. It is possible that results could be improved by capturing the conditional probabilities.

While the OLS results are more accurate on the aggregate level, there are additional benefits when using response mapping as opposed to mapping directly onto a preference-based score. Firstly, the predictions from the PPOMs can be used in conjunction with alternative preference weights to generate country specific EQ-5D scores. Secondly, the EQ-5D index score is an aggregate measure of five different aspects of health. Predicting the overall mean EQ-5D can mask changes in particular health dimensions and response mapping can provide additional information that would be lost at the summary level. The magnitude and direction of the coefficients in the PPOMs differed between equations for some health dimensions, reflecting the changes in the relationships when moving from one level of a health dimension to another and it would be difficult to capture these relationships in an OLS regression.

In summary, while the results presented here are promising, additional research exploring methods to improve the techniques used in responses mapping could improve results, increasing confidence in the predicted values when used to inform policy decision making of cost-effectiveness interventions and when used to explore potential differences in health care providers.

## Abbreviations

CVD: Cardiovascular disease; HRQoL: Health related quality of life; HSE: Health survey for England; LLTI: Limiting long term illnesses; MAE: Mean absolute errors; MSE: Mean squared error; OLM: Ordered logistic model; OLS: Ordinary least squares; PPOM: Partial proportional odds model; QALY: Quality adjusted life year; RMSE: Root mean squared error.

## Competing interests

The authors declare that they have no competing interests.

## Authors' contributions

RA designed the study, acquired the data, conducted the analyses, interpreted the data, and drafted the manuscript. BK contributed to both the data analyses and manuscript. BvH contributed to the design of the study and the manuscript, and provided support with the analyses and data interpretation. JEB participated in the design of the study and contributed to the manuscript. All authors read and approved the final manuscript.

## Supplementary Material

Additional file 1 Descriptive data for the explanatory variables. Click here for file

Additional file 2 Coefficients for additional models. Click here for file

## Health Survey for England data

The Health Survey for England is commissioned by the Department of Health and conducted by the Joint Health Survey Unit of National Centre for Social Research and Department of Epidemiology and Public Health at University College London. The original data creators, depositors or copyright holders, the funders of the Data Collections (if different) and the UK Data Archive bear no responsibility for the analysis presented here.
