# Supplementary material for: Predicting preference-based utility values using partial proportional odds models
Source: BMC Res Notes. 2014 Jul 8;7:438. doi: 10.1186/1756-0500-7-438 (PMC4118278; doi:10.1186/1756-0500-7-438)
Supplement: Additional file 1 — Descriptive data for the explanatory variables. [file 1756-0500-7-438-S1.docx]

APPENDIX A Descriptive data for the explanatory variables

| Survey Year | 2003 | 2004 | 2005 | 2006 | 2008 | 2003 | 2004 | 2005 | 2006 | 2008 | 2003 | 2004 | 2005 | 2006 | 2008 |
| --- | --- | --- | --- | --- | --- | --- | --- | --- | --- | --- | --- | --- | --- | --- | --- |
|  | CVD (n=7,998) | | | | | Diabetes (n=4,513) | | | | | Mental Health Conditions (n=1,901) | | | | |
| n | 1779 | 824 | 1694 | 1720 | 1,981 | 867 | 492 | 885 | 1031 | 1238 | 448 | 193 | 311 | 441 | 508 |
| EQ-5D (mean) | 0.740 | 0.719 | 0.720 | 0.716 | 0.723 | 0.754 | 0.736 | 0.750 | 0.746 | 0.725 | 0.597 | 0.590 | 0.625 | 0.597 | 0.586 |
| EQ-5D (sd) | 0.281 | 0.301 | 0.291 | 0.307 | 0.297 | 0.290 | 0.301 | 0.291 | 0.295 | 0.308 | 0.339 | 0.333 | 0.301 | 0.342 | 0.339 |
| Age (mean, years) | 63.8 | 65.0 | 68.7 | 65.1 | 65.1 | 59.9 | 60.6 | 65.2 | 60.6 | 61.6 | 47.5 | 47.3 | 51.1 | 45.8 | 46.0 |
| Male (%) | 48 | 45 | 49 | 48 | 49 | 42 | 39 | 41 | 42 | 40 | 40 | 38 | 37 | 40 | 38 |
| Education (%) | | | | | | | | | | | | | | | |
| No formal qualification [base] | 10 | 13 | 10 | 12 | 11 | 10 | 11 | 11 | 14 | 12 | 33 | 34 | 41 | 34 | 31 |
| GCSE or equivalent | 19 | 17 | 16 | 20 | 19 | 21 | 22 | 19 | 17 | 20 | 11 | 12 | 9 | 11 | 12 |
| A level or equivalent | 30 | 24 | 24 | 25 | 25 | 30 | 25 | 20 | 26 | 27 | 19 | 21 | 20 | 23 | 26 |
| Degree or equivalent | 41 | 46 | 50 | 44 | 45 | 39 | 42 | 50 | 43 | 40 | 38 | 34 | 30 | 32 | 31 |
| Deprivation (%) | | | | | | | | | | | | | | | |
| Least deprived [base] | 37.3 | 39.2 | 40.8 | 44.8 | 38.8 | 33.2 | 39.7 | 39.1 | 39.6 | 39.2 | 34.8 | 36.3 | 39.6 | 37.9 | 32.3 |
| A little deprived | 24.1 | 21.8 | 20.3 | 18.7 | 21.2 | 24.6 | 21.8 | 21.5 | 19.8 | 19.4 | 13.2 | 14.0 | 15.8 | 13.4 | 13.0 |
| Very deprived | 20.0 | 19.7 | 21.4 | 18.8 | 19.4 | 21.8 | 17.9 | 20.6 | 19.8 | 19.1 | 23.9 | 23.3 | 21.9 | 23.1 | 25.2 |
| Most deprived | 18.6 | 19.3 | 17.6 | 17.8 | 20.7 | 20.5 | 20.7 | 18.9 | 20.8 | 22.4 | 28.1 | 26.4 | 22.8 | 25.6 | 29.5 |
| General Health (%) |  |  |  |  |  |  |  |  |  |  |  |  |  |  |  |
| GHVG | 10.1 | 7.8 | 9.3 | 8.4 | 9.1 | 10.6 | 10.4 | 8.3 | 9.5 | 10.2 | 6.5 | 7.3 | 6.4 | 5.2 | 8.3 |
| GHG | 33.5 | 36.2 | 34.5 | 34.8 | 35.2 | 33.5 | 32.5 | 35.8 | 34.9 | 34.0 | 27.0 | 31.6 | 31.8 | 30.4 | 26.4 |
| Fair [base] | 38.5 | 37.3 | 37.4 | 36.9 | 37.6 | 38.8 | 38.6 | 37.6 | 36.9 | 35.9 | 40.4 | 31.6 | 35.4 | 41.0 | 35.0 |
| GHB | 13.8 | 14.1 | 13.6 | 14.8 | 13.8 | 11.4 | 13.2 | 12.9 | 14.6 | 14.7 | 21.0 | 22.8 | 20.6 | 18.4 | 21.7 |
| GHVB | 4.3 | 4.7 | 5.3 | 5.2 | 4.3 | 5.8 | 5.3 | 5.4 | 4.2 | 5.2 | 5.1 | 6.7 | 5.8 | 5.0 | 8.7 |
| Acute sickness (%) | | | | | | | | | | | | | | | |
| 0 days [base] | 79.4 | 80.3 | 78.2 | 77.1 | 78.8 | 78.4 | 78.7 | 81.0 | 80.0 | 79.5 | 71.7 | 71.0 | 70.1 | 69.2 | 71.3 |
| <6/14 days [Sick 1] | 3.8 | 3.4 | 3.1 | 3.4 | 4.2 | 3.8 | 4.5 | 3.3 | 3.4 | 4.0 | 5.6 | 4.2 | 7.1 | 6.1 | 6.1 |
| <13/14 days [Sick 2] | 4.9 | 3.3 | 4.4 | 5.4 | 3.7 | 5.0 | 3.9 | 4.1 | 4.4 | 3.8 | 7.8 | 10.9 | 6.4 | 7.7 | 7.3 |
| 14/14 days [Sick 3] | 11.9 | 13.0 | 14.2 | 14.1 | 13.2 | 12.8 | 13.0 | 11.6 | 12.2 | 12.7 | 15.0 | 14.0 | 16.4 | 17.0 | 15.4 |

Descriptive data for the explanatory variables (continued)

|  | 2003 | 2004 | 2005 | 2006 | 2008 | 2003 | 2004 | 2005 | 2006 | 2008 | 2003 | 2004 | 2005 | 2006 | 2008 |
| --- | --- | --- | --- | --- | --- | --- | --- | --- | --- | --- | --- | --- | --- | --- | --- |
|  | Musculoskeletal (n=11,290) | | | | | Nervous System (n=2,236 | | | | | Respiratory (n=5,110) | | | | |
| n | 2,793 | 1290 | 2,184 | 2,449 | 2,574 | 561 | 250 | 348 | 503 | 574 | 1231 | 599 | 891 | 1116 | 1273 |
| EQ-5D (mean) | 0.652 | 0.646 | 0.644 | 0.634 | 0.610 | 0.662 | 0.636 | 0.645 | 0.650 | 0.641 | 0.784 | 0.758 | 0.746 | 0.769 | 0.759 |
| EQ-5D (sd) | 0.300 | 0.309 | 0.296 | 0.312 | 0.317 | 0.327 | 0.361 | 0.315 | 0.339 | 0.340 | 0.276 | 0.309 | 0.300 | 0.303 | 0.304 |
| Age (mean, years) | 57.6 | 58.8 | 64.3 | 59.6 | 60.2 | 49.9 | 51.7 | 56.5 | 52.2 | 53.1 | 48.4 | 50.7 | 56.9 | 50.1 | 50.6 |
| Male (%) | 42 | 39 | 41 | 42 | 40 | 36 | 34 | 39 | 38 | 35 | 47 | 44 | 45 | 45 | 47 |
| Education (%) | | | | | | | | | | | | | | | |
| No formal qualification [base] | 37 | 42 | 45 | 41 | 43 | 14 | 15 | 11 | 16 | 13 | 29 | 30 | 37 | 30 | 30 |
| GCSE or equivalent | 10 | 11 | 10 | 13 | 11 | 24 | 24 | 23 | 21 | 24 | 14 | 16 | 14 | 16 | 15 |
| A level or equivalent | 21 | 22 | 19 | 21 | 19 | 33 | 28 | 31 | 28 | 30 | 28 | 28 | 26 | 28 | 29 |
| Degree or equivalent | 32 | 24 | 26 | 26 | 27 | 29 | 33 | 34 | 35 | 33 | 29 | 26 | 23 | 26 | 26 |
| Deprivation (%) | | | | | | | | | | | | | | | |
| Least deprived [base] | 39.2 | 39.9 | 41.0 | 42.9 | 40.1 | 30.0 | 34.8 | 35.8 | 36.4 | 34.4 | 38.8 | 36.1 | 40.2 | 41.9 | 38.8 |
| A little deprived | 22.0 | 19.7 | 20.3 | 19.7 | 19.2 | 19.8 | 17.6 | 18.7 | 19.3 | 17.9 | 20.5 | 21.2 | 19.8 | 17.7 | 18.9 |
| Very deprived | 20.2 | 21.2 | 21.1 | 19.7 | 20.1 | 22.1 | 21.2 | 22.7 | 18.7 | 18.1 | 20.3 | 22.0 | 21.2 | 19.7 | 19.9 |
| Most deprived | 18.6 | 19.3 | 17.6 | 17.8 | 20.7 | 28.1 | 26.4 | 22.8 | 25.6 | 29.5 | 20.5 | 20.7 | 18.9 | 20.8 | 22.4 |
| General Health (%) | | | | | | | | | | | | | | | |
| GHVG | 12.5 | 14.3 | 10.9 | 11.8 | 10.3 | 11.2 | 11.2 | 10.3 | 10.9 | 12.2 | 15.7 | 15.5 | 13.4 | 15.4 | 16.3 |
| GHG | 35.9 | 35.5 | 34.8 | 34.5 | 34.5 | 34.2 | 33.2 | 30.2 | 29.8 | 30.5 | 38.2 | 38.6 | 34.8 | 36.7 | 36.1 |
| Fair [base] | 34.3 | 32.0 | 35.9 | 34.8 | 35.6 | 31.9 | 28.0 | 35.4 | 32.4 | 30.5 | 29.0 | 28.9 | 31.2 | 29.6 | 27.9 |
| GHB | 12.6 | 13.9 | 13.1 | 14.7 | 14.7 | 15.5 | 20.8 | 17.8 | 21.3 | 19.2 | 13.1 | 12.7 | 13.8 | 13.0 | 13.8 |
| GHVB | 4.7 | 4.3 | 5.2 | 4.3 | 4.9 | 7.1 | 6.8 | 6.3 | 5.6 | 7.7 | 4.1 | 4.3 | 6.9 | 5.3 | 5.9 |
| Acute sickness (%) | | | | | | | | | | | | | | | |
| 0 days [Base] | 74.3 | 75.4 | 74.7 | 72.6 | 72.9 | 71.5 | 65.6 | 71.0 | 70.8 | 72.7 | 79.1 | 76.8 | 77.4 | 76.7 | 76.0 |
| <6/14 days [Sick 1] | 4.7 | 4.3 | 4.4 | 4.7 | 5.5 | 5.9 | 6.4 | 5.2 | 6.0 | 5.9 | 4.1 | 5.2 | 4.5 | 4.5 | 4.9 |
| <13/14 days [Sick 2] | 6.4 | 3.8 | 4.9 | 6.2 | 5.2 | 6.6 | 8.4 | 6.9 | 6.2 | 5.4 | 6.3 | 4.8 | 5.5 | 5.6 | 5.8 |
| 14/14 days [Sick 3] | 14.7 | 16.4 | 15.9 | 16.4 | 16.4 | 16.0 | 19.6 | 17.0 | 17.1 | 16.0 | 10.6 | 13.2 | 12.6 | 13.3 | 13.3 |
