# Supplementary material for: Predicting preference-based utility values using partial proportional odds models
Source: BMC Res Notes. 2014 Jul 8;7:438. doi: 10.1186/1756-0500-7-438 (PMC4118278; doi:10.1186/1756-0500-7-438)
Supplement: Additional file 2 — Coefficients for additional models. [file 1756-0500-7-438-S2.docx]

APPENDIX B Coefficients for additional models

Model coefficients for the LLTI Diabetes

| Method | OLS | | PPOM^a^ | | PPOM | | PPOM | | PPOM | | PPOM | |
| --- | --- | --- | --- | --- | --- | --- | --- | --- | --- | --- | --- | --- |
| DV | EQ-5D | | MOBILITY | | SELF CARE | | USUAL ACTIVITIES | | PAIN | | ANXIETY/DEPRESSION | |
|  | Beta | SE | Beta | SE | Beta | SE | Beta | seSE | Beta | SE | Beta | SE |
| n | 4513 |  | 4513 |  | 4513 |  | 4513 |  | 4513 |  | 4513 | 0 |
| Age | -0.0011 | 0.001 | -0.0295 | 0.023 | 0.0083 | 0.030 | -0.0530 | 0.020* | 0.0710 | 0.016*** | 0.0321 | 0.016* |
|  |  |  | -0.0295 | 0.023 | -0.1797 | 0.069** | -0.0320 | 0.022 | 0.0710 | 0.016*** | 0.0321 | 0.016* |
| Age2 | 0.0000 | 0.000 | 0.0007 | 0.000** | 0.0000 | 0.000 | 0.0005 | 0.000** | -0.0005 | 0.000*** | -0.0004 | 0.000** |
|  |  |  | 0.0001 | 0.000 | 0.0015 | 0.001** | 0.0005 | 0.000** | -0.0005 | 0.000*** | -0.0006 | 0.000*** |
| Sex | -0.0347 | 0.007*** | 0.0605 | 0.098 | -0.0247 | 0.119 | -0.0467 | 0.091 | 0.2726 | 0.070*** | 0.4194 | 0.077*** |
|  |  |  | 0.0605 | 0.098 | -0.0247 | 0.119 | -0.0467 | 0.091 | 0.2726 | 0.070*** | 0.4194 | 0.077*** |
| GCSE | 0.0297 | 0.010** | -0.1100 | 0.176 | -0.0625 | 0.259 | -0.0331 | 0.176 | -0.1386 | 0.129 | -0.2540 | 0.142 |
|  |  |  | -0.1100 | 0.176 | -0.0625 | 0.259 | -0.0331 | 0.176 | -0.8944 | 0.366* | -0.2540 | 0.142 |
| A level | 0.0022 | 0.010 | -0.0211 | 0.140 | -0.2431 | 0.180 | 0.1625 | 0.134 | 0.0330 | 0.100 | -0.1056 | 0.110 |
|  |  |  | -0.0211 | 0.140 | -0.2431 | 0.180 | 0.1625 | 0.134 | 0.0330 | 0.100 | -0.1056 | 0.110 |
| Degree | 0.0102 | 0.009 | -0.2241 | 0.122 | 0.0107 | 0.150 | 0.1424 | 0.115 | -0.0062 | 0.088 | -0.1046 | 0.096 |
|  |  |  | -0.2241 | 0.122 | 0.0107 | 0.150 | 0.1424 | 0.115 | -0.0062 | 0.088 | -0.1046 | 0.096 |
| Sick 1 | -0.0485 | 0.020* | -0.0439 | 0.231 | 0.2382 | 0.254 | 0.4877 | 0.201* | 0.4364 | 0.177* | -0.0694 | 0.183 |
|  |  |  | -0.0439 | 0.231 | 0.2382 | 0.254 | 0.4877 | 0.201* | 0.4364 | 0.177* | -0.0694 | 0.183 |
| Sick 2 | -0.1164 | 0.021*** | 0.2314 | 0.227 | 0.3323 | 0.208 | 0.3113 | 0.184 | 0.5490 | 0.168** | 0.2716 | 0.162 |
|  |  |  | 0.2314 | 0.227 | 0.3323 | 0.208 | 0.3113 | 0.184 | 0.5490 | 0.168** | 0.2716 | 0.162 |
| Sick 3 | -0.1663 | 0.014*** | 0.3385 | 0.157* | 0.2494 | 0.138 | 0.8301 | 0.122*** | 0.5319 | 0.113*** | 0.0996 | 0.109 |
|  |  |  | 0.3385 | 0.157* | 0.2494 | 0.138 | 0.8301 | 0.122*** | 0.5319 | 0.113*** | 0.0996 | 0.109 |
| A little deprived | 0.0188 | 0.008* | -0.0317 | 0.129 | -0.2290 | 0.182 | -0.0971 | 0.127 | -0.0828 | 0.092 | 0.0952 | 0.104 |
|  |  |  | -0.0317 | 0.129 | -0.2290 | 0.182 | -0.0971 | 0.127 | -0.0828 | 0.092 | 0.0952 | 0.104 |
| Very deprived | -0.0085 | 0.010 | 0.2168 | 0.127 | 0.0110 | 0.155 | -0.0149 | 0.119 | 0.0309 | 0.092 | 0.0516 | 0.100 |
|  |  |  | 0.2168 | 0.127 | 0.0110 | 0.155 | -0.0149 | 0.119 | 0.0309 | 0.092 | 0.0516 | 0.100 |
| Most deprived | -0.0449 | 0.011*** | 0.1084 | 0.136 | 0.0615 | 0.150 | 0.1605 | 0.121 | 0.1775 | 0.098 | 0.1649 | 0.102 |
|  |  |  | 0.1084 | 0.136 | 0.0615 | 0.150 | 0.1605 | 0.121 | 0.1775 | 0.098 | 0.1649 | 0.102 |
| GHVG | 0.1508 | 0.009*** | -1.0833 | 0.217*** | 0.0141 | 0.414 | -1.2656 | 0.269*** | -1.0066 | 0.142*** | -1.1595 | 0.187*** |
|  |  |  | 1.6397 | 1.108 | 0.0141 | 0.414 | 0.2204 | 0.637 | -1.0066 | 0.142*** | -1.1595 | 0.187*** |
| GHG | 0.1104 | 0.007*** | -0.8168 | 0.113*** | -0.2350 | 0.213 | -0.7754 | 0.122*** | -0.3364 | 0.083*** | -0.6658 | 0.099*** |
|  |  |  | -0.8168 | 0.113*** | 0.7969 | 0.511 | -0.7754 | 0.122*** | -0.3364 | 0.083*** | -0.6658 | 0.099*** |
| GHB | -0.2497 | 0.015*** | 0.3711 | 0.154* | 0.3677 | 0.141** | 0.7778 | 0.127*** | 0.6619 | 0.120*** | 0.6448 | 0.108*** |
|  |  |  | 0.3711 | 0.154* | 0.3677 | 0.141** | 0.7778 | 0.127*** | 0.6619 | 0.120*** | 0.6448 | 0.108*** |
| GHVB | -0.4211 | 0.025*** | 0.8129 | 0.275** | 0.5493 | 0.192** | 0.5684 | 0.273* | 0.1843 | 0.309 | 0.8769 | 0.159*** |
|  |  |  | 0.8129 | 0.275** | 0.5493 | 0.192** | 1.4705 | 0.213*** | 1.0981 | 0.184*** | 0.8769 | 0.159*** |
| Year2004 | -0.0147 | 0.013 | 0.0430 | 0.178 | 0.1143 | 0.220 | -0.0714 | 0.169 | 0.1673 | 0.128 | -0.0485 | 0.136 |
|  |  |  | 0.0430 | 0.178 | 0.1143 | 0.220 | -0.0714 | 0.169 | 0.1673 | 0.128 | -0.0485 | 0.136 |
| Year2005 | 0.0076 | 0.010 | -0.1439 | 0.152 | -0.0569 | 0.192 | 0.0008 | 0.143 | 0.1562 | 0.109 | -0.4837 | 0.123*** |
|  |  |  | -0.1439 | 0.152 | -0.0569 | 0.192 | 0.0008 | 0.143 | 0.1562 | 0.109 | -0.4837 | 0.123*** |
| Year2006 | -0.0079 | 0.010 | -0.0636 | 0.148 | -0.0089 | 0.182 | 0.0458 | 0.138 | 0.0542 | 0.106 | -0.1146 | 0.112 |
|  |  |  | -0.0636 | 0.148 | -0.0089 | 0.182 | 0.0458 | 0.138 | 0.0542 | 0.106 | -0.1146 | 0.112 |
| Year2008 | -0.0200 | 0.010* | 0.0712 | 0.141 | 0.0489 | 0.173 | -0.0019 | 0.131 | 0.1769 | 0.101 | 0.0209 | 0.106 |
|  |  |  | 0.0712 | 0.141 | 0.0489 | 0.173 | -0.0019 | 0.131 | 0.1769 | 0.101 | 0.0209 | 0.106 |
| Mobility |  |  |  |  | 1.6896 | 0.206*** | 2.0920 | 0.111*** | 1.7655 | 0.101*** | -0.0785 | 0.106 |
|  |  |  |  |  | 1.6896 | 0.206*** | 1.0983 | 0.318** | 1.7655 | 0.101*** | -0.0785 | 0.106 |
| Self care |  |  | 1.1147 | 0.185*** |  |  | 1.8700 | 0.202*** | 0.4741 | 0.114*** | 0.4451 | 0.103*** |
|  |  |  | 1.1147 | 0.185*** |  |  | 1.3436 | 0.157*** | 0.4741 | 0.114*** | 0.4451 | 0.103*** |
| Usual act |  |  | 1.9263 | 0.107*** | 1.6365 | 0.130*** |  |  | 1.1830 | 0.093*** | 0.3435 | 0.091*** |
|  |  |  | 1.9263 | 0.107*** | 1.6365 | 0.130*** |  |  | 1.1830 | 0.093*** | 0.9970 | 0.152*** |
| Pain |  |  | 1.7411 | 0.103*** | 0.7014 | 0.119*** | 1.3191 | 0.109*** |  |  | 0.3952 | 0.076*** |
|  |  |  | -0.1521 | 0.430 | -0.1781 | 0.280 | 0.8618 | 0.157*** |  |  | 0.3952 | 0.076*** |
| Anxiety |  |  | -0.1469 | 0.100 | 0.5005 | 0.097*** | 0.4498 | 0.084*** | 0.3707 | 0.072*** |  |  |
|  |  |  | -0.1469 | 0.100 | 0.5005 | 0.097*** | 0.4498 | 0.084*** | 0.3707 | 0.072*** |  |  |
| Constant | 0.9368 | 0.039*** | -7.9195 | 0.792*** | -10.4999 | 1.051*** | -7.5167 | 0.698*** | -7.2615 | 0.527*** | -3.3250 | 0.524*** |
|  |  |  | -9.5226 | 1.495*** | -6.5549 | 2.245** | -9.9822 | 1.027*** | -11.8086 | 0.562*** | -6.9312 | 0.611*** |

^a^As the PPOMs comprise of two equations, each explanatory variable has two rows with two Beta coefficients (and corresponding SE). *p<0.05; **p<0.01; ***p<0.001

Model coefficients for the LLTI Respiratory

| Method | OLS | | PPOM^a^ | | PPOM | | PPOM | | PPOM | | PPOM | |
| --- | --- | --- | --- | --- | --- | --- | --- | --- | --- | --- | --- | --- |
| DV | EQ-5D | | MOBILITY | | SELF CARE | | USUAL ACTIVITIES | | PAIN | | ANXIETY/DEPRESSION | |
|  | Beta | SE | Beta | SE | Beta | SE | Beta | SE | Beta | SE | Beta | SE |
| n | 5110 |  | 5110 | | 5110 | | 5110 | | 5110 | | 5110 | |
| Age | -0.0019 | 0.001* | 0.0359 | 0.017* | -0.0106 | 0.023 | -0.0323 | 0.014* | 0.0659 | 0.010*** | 0.0050 | 0.010 |
|  |  |  | -0.0759 | 0.031* | -0.1476 | 0.057** | -0.0138 | 0.016 | 0.0518 | 0.012*** | -0.0135 | 0.011 |
| Age2 | 0.0000 | 0.000 | 0.0001 | 0.000 | 0.0002 | 0.000 | 0.0003 | 0.000** | -0.0005 | 0.000*** | -0.0002 | 0.000 |
|  |  |  | 0.0001 | 0.000 | 0.0014 | 0.000** | 0.0003 | 0.000** | -0.0005 | 0.000*** | -0.0002 | 0.000 |
| Sex | -0.0366 | 0.006*** | 0.3175 | 0.102** | -0.0902 | 0.121 | -0.0795 | 0.089 | 0.2375 | 0.067*** | 0.3800 | 0.070*** |
|  |  |  | 0.3175 | 0.102** | -0.0902 | 0.121 | -0.0795 | 0.089 | 0.2375 | 0.067*** | 0.3800 | 0.070*** |
| GCSE | 0.0597 | 0.010*** | -0.5961 | 0.187** | -0.7089 | 0.323* | 0.0170 | 0.177 | -0.2705 | 0.119* | -0.1266 | 0.130 |
|  |  |  | 1.9059 | 1.188 | -0.7089 | 0.323* | 0.0170 | 0.177 | -0.2705 | 0.119* | -1.0867 | 0.434* |
| A level | 0.0451 | 0.010*** | -0.2816 | 0.143* | -0.1322 | 0.175 | 0.1828 | 0.128 | -0.1584 | 0.098 | -0.2395 | 0.104* |
|  |  |  | -0.2816 | 0.143* | -0.1322 | 0.175 | 0.1828 | 0.128 | -0.1584 | 0.098 | -0.2395 | 0.104* |
| Degree | 0.0415 | 0.010*** | -0.3393 | 0.129** | -0.0914 | 0.150 | 0.1636 | 0.113 | -0.1235 | 0.090 | -0.0112 | 0.095 |
|  |  |  | -0.3393 | 0.129** | -1.6300 | 0.768* | 0.1636 | 0.113 | -0.1235 | 0.090 | -0.4191 | 0.193* |
| Sick 1 | -0.0669 | 0.017*** | 0.1919 | 0.209 | 0.1419 | 0.242 | 0.5067 | 0.180** | 0.3894 | 0.148** | 0.2122 | 0.151 |
|  |  |  | 0.1919 | 0.209 | 0.1419 | 0.242 | 0.5067 | 0.180** | 0.3894 | 0.148** | 0.2122 | 0.151 |
| Sick 2 | -0.0987 | 0.017*** | 0.3012 | 0.201 | 0.3498 | 0.199 | 0.5290 | 0.159** | 0.2868 | 0.138* | 0.2954 | 0.139* |
|  |  |  | 0.3012 | 0.201 | 0.3498 | 0.199 | 0.5290 | 0.159** | 0.2868 | 0.138* | 0.7671 | 0.222** |
| Sick 3 | -0.1400 | 0.013*** | 0.3735 | 0.154* | 0.4588 | 0.135** | 0.6716 | 0.118*** | 0.4060 | 0.106*** | 0.1583 | 0.103 |
|  |  |  | 0.3735 | 0.154* | 0.4588 | 0.135** | 0.6716 | 0.118*** | 0.4060 | 0.106*** | 0.1583 | 0.103 |
| A little deprived | 0.0133 | 0.007 | 0.1559 | 0.142 | -0.1661 | 0.205 | -0.1639 | 0.133 | -0.0696 | 0.099 | 0.0335 | 0.101 |
|  |  |  | 0.1559 | 0.142 | 1.6165 | 0.458*** | -0.1639 | 0.133 | -0.6481 | 0.233** | 0.0335 | 0.101 |
| Very deprived | -0.0068 | 0.008 | 0.4277 | 0.135** | 0.0800 | 0.159 | 0.0008 | 0.117 | -0.0343 | 0.090 | -0.0213 | 0.094 |
|  |  |  | 0.4277 | 0.135** | 0.0800 | 0.159 | 0.0008 | 0.117 | -0.0343 | 0.090 | -0.0213 | 0.094 |
| Most deprived | -0.0185 | 0.009* | 0.1346 | 0.139 | 0.2972 | 0.149* | -0.1270 | 0.116 | 0.0079 | 0.091 | 0.1536 | 0.092 |
|  |  |  | 0.1346 | 0.139 | 0.2972 | 0.149* | -0.1270 | 0.116 | 0.0079 | 0.091 | 0.1536 | 0.092 |
| GHVG | 0.1467 | 0.008*** | -1.5759 | 0.246*** | -0.0449 | 0.495 | -1.4508 | 0.251*** | -1.1310 | 0.128*** | -1.0270 | 0.144*** |
|  |  |  | 2.8481 | 1.275* | 2.1995 | 1.072* | 0.3925 | 0.546 | -1.1310 | 0.128*** | -3.1854 | 1.012** |
| GHG | 0.1080 | 0.007*** | -0.8287 | 0.120*** | -0.2882 | 0.232 | -0.7724 | 0.121*** | -0.4739 | 0.085*** | -0.5050 | 0.094*** |
|  |  |  | -0.8287 | 0.120*** | -0.2882 | 0.232 | -0.7724 | 0.121*** | -0.4739 | 0.085*** | -1.3962 | 0.307*** |
| GHB | -0.2459 | 0.014*** | 0.5083 | 0.149** | 0.2903 | 0.144* | 0.7139 | 0.124*** | 0.3341 | 0.149* | 0.3634 | 0.106** |
|  |  |  | 0.5083 | 0.149** | 0.2903 | 0.144* | 0.7139 | 0.124*** | 0.8625 | 0.142*** | 0.3634 | 0.106** |
| GHVB | -0.4152 | 0.022*** | 1.1891 | 0.317*** | 0.5715 | 0.183** | 0.8458 | 0.265** | 0.9116 | 0.171*** | 0.4878 | 0.160** |
|  |  |  | 1.1891 | 0.317*** | 0.5715 | 0.183** | 1.4434 | 0.199*** | 0.9116 | 0.171*** | 0.9744 | 0.211*** |
| Year2004 | -0.0177 | 0.011 | -0.4802 | 0.178** | 0.1825 | 0.218 | -0.0555 | 0.154 | 0.2117 | 0.115 | 0.1195 | 0.120 |
|  |  |  | 3.0156 | 1.378* | 0.1825 | 0.218 | -0.0555 | 0.154 | 0.2117 | 0.115 | 0.1195 | 0.120 |
| Year2005 | 0.0002 | 0.010 | -0.0829 | 0.153 | 0.2557 | 0.181 | -0.1282 | 0.134 | -0.0083 | 0.104 | -0.1773 | 0.110 |
|  |  |  | -0.0829 | 0.153 | 0.2557 | 0.181 | -0.1282 | 0.134 | -0.0083 | 0.104 | -0.1773 | 0.110 |
| Year2006 | -0.0007 | 0.009 | -0.2629 | 0.148 | 0.1667 | 0.180 | -0.1631 | 0.129 | 0.0182 | 0.097 | 0.0135 | 0.101 |
|  |  |  | -0.2629 | 0.148 | 0.1667 | 0.180 | -0.1631 | 0.129 | 0.0182 | 0.097 | 0.0135 | 0.101 |
| Year2008 | -0.0068 | 0.008 | -0.2175 | 0.144 | 0.2673 | 0.171 | -0.2065 | 0.124 | 0.0683 | 0.094 | 0.0266 | 0.097 |
|  |  |  | 3.0286 | 1.203* | 0.2673 | 0.171 | -0.2065 | 0.124 | 0.0683 | 0.094 | 0.0266 | 0.097 |
| Mobility |  |  |  |  | 1.8121 | 0.228*** | 2.0071 | 0.113*** | 1.5184 | 0.101*** | -0.0472 | 0.106 |
|  |  |  |  |  | 1.8121 | 0.228*** | 1.1114 | 0.307*** | 1.5184 | 0.101*** | -0.0472 | 0.106 |
| Self care |  |  | 1.0241 | 0.233*** |  |  | 2.0717 | 0.235*** | 0.5976 | 0.116*** | 0.4427 | 0.104*** |
|  |  |  | 2.9655 | 0.678*** |  |  | 1.0152 | 0.156*** | 0.5976 | 0.116*** | 0.4427 | 0.104*** |
| Usual act |  |  | 1.8498 | 0.110*** | 1.4512 | 0.126*** |  |  | 0.9840 | 0.087*** | 0.5407 | 0.084*** |
|  |  |  | 1.8498 | 0.110*** | 1.4512 | 0.126*** |  |  | 0.9840 | 0.087*** | 0.5407 | 0.084*** |
| Pain |  |  | 1.4599 | 0.100*** | 0.8283 | 0.112*** | 1.1251 | 0.098*** |  |  | 0.4780 | 0.070*** |
|  |  |  | -0.8623 | 0.630 | 0.8283 | 0.112*** | 0.6245 | 0.147*** |  |  | 0.4780 | 0.070*** |
| Anxiety |  |  | -0.0558 | 0.097 | 0.4454 | 0.094*** | 0.5171 | 0.077*** | 0.4204 | 0.065*** |  |  |
|  |  |  | -0.0558 | 0.097 | 0.4454 | 0.094*** | 0.5171 | 0.077*** | 0.4204 | 0.065*** |  |  |
| Constant | 0.8912 | 0.023*** | -9.0677 | 0.604*** | -10.3481 | 0.821*** | -7.7596 | 0.503*** | -6.3650 | 0.350*** | -3.0816 | 0.334*** |
|  |  |  | -12.8365 | 2.411*** | -11.1800 | 1.717*** | -9.3234 | 0.795*** | -9.7108 | 0.474*** | -4.5951 | 0.433*** |

^a^As the PPOMs comprise of two equations, each explanatory variable has two rows with two Beta coefficients (and corresponding SE). *p<0.05; **p<0.01; ***p<0.001

Model coefficients for the LLTI CVD

| Method | OLS | | PPOM^a^ | | PPOM | | PPOM | | PPOM | | PPOM | |
| --- | --- | --- | --- | --- | --- | --- | --- | --- | --- | --- | --- | --- |
| DV | EQ-5D | | MOBILITY | | SELF CARE | | USUAL ACTIVITIES | | PAIN | | ANXIETY/DEPRESSION | |
|  | Beta | SE | Beta | SE | Beta | SE | Beta | SE | Beta | SE | Beta | SE |
| n | 7998 |  | 7998 |  | 7998 |  | 7998 |  | 7998 |  | 7998 |  |
| Age | 0.0006 | 0.001 | -0.0069 | 0.019 | -0.0153 | 0.025 | -0.0410 | 0.016* | 0.0554 | 0.013*** | -0.0374 | 0.012** |
|  |  |  | -0.0698 | 0.025** | -0.0153 | 0.025 | -0.0410 | 0.016* | 0.0554 | 0.013*** | -0.0374 | 0.012** |
| Age2 | 0.0000 | 0.000* | 0.0004 | 0.000** | 0.0002 | 0.000 | 0.0004 | 0.000** | -0.0004 | 0.000*** | 0.0002 | 0.000 |
|  |  |  | 0.0004 | 0.000** | 0.0002 | 0.000 | 0.0004 | 0.000** | -0.0005 | 0.000*** | 0.0000 | 0.000 |
| Sex | -0.0291 | 0.005*** | 0.1148 | 0.070 | -0.1954 | 0.082* | -0.0475 | 0.064 | 0.2955 | 0.051*** | 0.2860 | 0.056*** |
|  |  |  | 0.1148 | 0.070 | -0.1954 | 0.082* | -0.0475 | 0.064 | 0.2955 | 0.051*** | 0.2860 | 0.056*** |
| GCSE | 0.0427 | 0.008*** | -0.3168 | 0.130* | -0.2502 | 0.188 | 0.1643 | 0.125 | -0.3036 | 0.093** | -0.1739 | 0.107 |
|  |  |  | -0.3168 | 0.130* | -0.2502 | 0.188 | 0.1643 | 0.125 | -0.3036 | 0.093** | -0.1739 | 0.107 |
| A level | 0.0096 | 0.007 | -0.0344 | 0.100 | -0.1372 | 0.125 | 0.1875 | 0.093* | -0.1058 | 0.074 | -0.0568 | 0.082 |
|  |  |  | -0.0344 | 0.100 | -0.1372 | 0.125 | 0.1875 | 0.093* | -0.1058 | 0.074 | -0.0568 | 0.082 |
| Degree | 0.0119 | 0.007 | -0.2476 | 0.086** | 0.0366 | 0.102 | 0.1070 | 0.080 | 0.0038 | 0.064 | -0.1520 | 0.071* |
|  |  |  | -0.2476 | 0.086** | 0.0366 | 0.102 | 0.1070 | 0.080 | 0.0038 | 0.064 | -0.1520 | 0.071* |
| Sick 1 | -0.0804 | 0.016*** | 0.3727 | 0.177* | 0.1565 | 0.176 | 0.1432 | 0.146 | 0.4727 | 0.133*** | 0.2881 | 0.131* |
|  |  |  | 0.3727 | 0.177* | 0.1565 | 0.176 | 0.1432 | 0.146 | 0.4727 | 0.133*** | 0.2881 | 0.131* |
| Sick 2 | -0.0884 | 0.015*** | 0.1200 | 0.170 | 0.1064 | 0.154 | 0.3848 | 0.134** | 0.3612 | 0.122** | 0.4005 | 0.117** |
|  |  |  | 0.1200 | 0.170 | 0.1064 | 0.154 | 0.3848 | 0.134** | 0.3612 | 0.122** | 0.4005 | 0.117** |
| Sick 3 | -0.1282 | 0.010*** | 0.2276 | 0.110* | 0.1737 | 0.096 | 0.9577 | 0.107*** | 0.3383 | 0.080*** | 0.1741 | 0.078* |
|  |  |  | 0.2276 | 0.110* | 0.1737 | 0.096 | 0.5464 | 0.124*** | 0.3383 | 0.080*** | 0.1741 | 0.078* |
| A little deprived | 0.0125 | 0.006* | 0.0150 | 0.092 | -0.2653 | 0.127* | -0.1054 | 0.095 | -0.0577 | 0.068 | -0.0774 | 0.078 |
|  |  |  | 0.0150 | 0.092 | -0.2653 | 0.127* | 0.2417 | 0.163 | -0.0577 | 0.068 | -0.0774 | 0.078 |
| Very deprived | -0.0152 | 0.007* | 0.1478 | 0.093 | -0.0273 | 0.107 | 0.0931 | 0.084 | 0.0910 | 0.068 | -0.0074 | 0.074 |
|  |  |  | 0.1478 | 0.093 | -0.0273 | 0.107 | 0.0931 | 0.084 | 0.0910 | 0.068 | -0.0074 | 0.074 |
| Most deprived | -0.0441 | 0.008*** | 0.1028 | 0.100 | 0.2041 | 0.105 | 0.0508 | 0.088 | 0.1327 | 0.073 | 0.1056 | 0.076 |
|  |  |  | 0.1028 | 0.100 | 0.2041 | 0.105 | 0.0508 | 0.088 | 0.1327 | 0.073 | 0.1056 | 0.076 |
| GHVG | 0.1589 | 0.007*** | -1.1608 | 0.148*** | -0.0308 | 0.282 | -0.9548 | 0.165*** | -0.7588 | 0.102*** | -1.1654 | 0.147*** |
|  |  |  | -1.1608 | 0.148*** | -0.0308 | 0.282 | -0.9548 | 0.165*** | -0.7588 | 0.102*** | -1.1654 | 0.147*** |
| GHG | 0.1196 | 0.006*** | -0.8042 | 0.080*** | -0.3137 | 0.142* | -0.8663 | 0.085*** | -0.3574 | 0.063*** | -0.6079 | 0.074*** |
|  |  |  | -0.8042 | 0.080*** | 0.7621 | 0.395 | -0.2177 | 0.217 | -0.3574 | 0.063*** | -0.6079 | 0.074*** |
| GHB | -0.2461 | 0.011*** | 0.5798 | 0.121*** | 0.4873 | 0.097*** | 0.5857 | 0.090*** | 0.5290 | 0.124*** | 0.3257 | 0.081*** |
|  |  |  | -0.8538 | 0.574 | 0.4873 | 0.097*** | 0.5857 | 0.090*** | 0.9264 | 0.105*** | 0.9118 | 0.166*** |
| GHVB | -0.4118 | 0.019*** | 0.5849 | 0.228* | 0.5402 | 0.139*** | 0.7054 | 0.217** | 0.7287 | 0.262** | 0.5095 | 0.127*** |
|  |  |  | 0.5849 | 0.228* | 0.5402 | 0.139*** | 1.3356 | 0.155*** | 1.3254 | 0.141*** | 1.3291 | 0.197*** |
| Year2004 | -0.0148 | 0.010 | -0.1849 | 0.129 | 0.0780 | 0.155 | -0.1041 | 0.118 | 0.2785 | 0.094** | -0.0643 | 0.102 |
|  |  |  | -0.1849 | 0.129 | 0.0780 | 0.155 | -0.1041 | 0.118 | 0.2785 | 0.094** | -0.0643 | 0.102 |
| Year2005 | -0.0025 | 0.008 | -0.1386 | 0.102 | 0.1444 | 0.124 | -0.1642 | 0.095 | 0.2503 | 0.077** | -0.2763 | 0.085** |
|  |  |  | -0.1386 | 0.102 | 0.1444 | 0.124 | -0.1642 | 0.095 | 0.2503 | 0.077** | -0.2763 | 0.085** |
| Year2006 | -0.0114 | 0.008 | -0.0362 | 0.103 | 0.1514 | 0.123 | -0.1143 | 0.094 | 0.0921 | 0.076 | -0.0109 | 0.081 |
|  |  |  | -0.0362 | 0.103 | 0.6912 | 0.274* | -0.1143 | 0.094 | 0.0921 | 0.076 | -0.0109 | 0.081 |
| Year2008 | -0.0126 | 0.007 | -0.1565 | 0.100 | 0.1706 | 0.119 | -0.1170 | 0.091 | 0.2104 | 0.073** | -0.0306 | 0.079 |
|  |  |  | -0.1565 | 0.100 | 0.1706 | 0.119 | -0.1170 | 0.091 | 0.2104 | 0.073** | -0.0306 | 0.079 |
| Mobility |  |  |  |  | 1.7264 | 0.155*** | 2.0836 | 0.079*** | 1.6857 | 0.072*** | -0.0521 | 0.078 |
|  |  |  |  |  | 1.7264 | 0.155*** | 1.4347 | 0.225*** | 1.6857 | 0.072*** | -0.0521 | 0.078 |
| Self care |  |  | 1.3030 | 0.147*** |  |  | 1.9169 | 0.143*** | -0.0651 | 0.125 | 0.5063 | 0.073*** |
|  |  |  | 1.3030 | 0.147*** |  |  | 1.4380 | 0.111*** | 0.6226 | 0.090*** | 0.5063 | 0.073*** |
| Usual act |  |  | 1.8909 | 0.076*** | 1.5525 | 0.088*** |  |  | 0.9259 | 0.064*** | 0.3580 | 0.063*** |
|  |  |  | 1.8909 | 0.076*** | 2.2272 | 0.250*** |  |  | 0.9259 | 0.064*** | 0.3580 | 0.063*** |
| Pain |  |  | 1.6121 | 0.072*** | 0.6477 | 0.080*** | 1.1042 | 0.075*** |  |  | 0.4950 | 0.055*** |
|  |  |  | -0.3590 | 0.330 | -0.2025 | 0.205 | 0.5759 | 0.105*** |  |  | 0.4950 | 0.055*** |
| Anxiety |  |  | -0.1030 | 0.074 | 0.5283 | 0.069*** | 0.4617 | 0.072*** | 0.4637 | 0.053*** |  |  |
|  |  |  | -0.1030 | 0.074 | 0.5283 | 0.069*** | 0.2298 | 0.093* | 0.4637 | 0.053*** |  |  |
| Constant | 0.8512 | 0.038*** | -8.1159 | 0.657*** | -9.5902 | 0.893*** | -7.4776 | 0.572*** | -6.0547 | 0.449*** | -1.3800 | 0.410** |
|  |  |  | -7.9937 | 1.505*** | -13.2630 | 1.139*** | -8.4912 | 0.705*** | -10.9879 | 0.505*** | -4.1301 | 0.447*** |

^a^As the PPOMs comprise of two equations, each explanatory variable has two rows with two Beta coefficients (and corresponding SE). *p<0.05; **p<0.01; ***p<0.001

Model coefficients for the LLTI Mental health disorders

| Method | OLS | | PPOM^a^ | | PPOM | | PPOM | | PPOM | | PPOM | |
| --- | --- | --- | --- | --- | --- | --- | --- | --- | --- | --- | --- | --- |
| DV | EQ-5D | | MOBILITY | | SELF CARE | | USUAL ACTIVITIES | | PAIN | | ANXIETY/DEPRESSION | |
|  | Beta | SE | Beta | SE | Beta | SE | Beta | SE | Beta | SE | Beta | SE |
| n | 1901 |  | 1901 |  | 1901 |  | 1901 |  | 1901 |  | 1901 |  |
| Age | -0.0015 | 0.002 | -0.0094 | 0.023 | -0.0693 | 0.025** | -0.0290 | 0.019 | 0.0677 | 0.017*** | 0.0436 | 0.015** |
|  |  |  | -0.0094 | 0.023 | -0.0693 | 0.025** | -0.0024 | 0.023 | 0.0677 | 0.017*** | 0.0436 | 0.015** |
| Age2 | 0.0000 | 0.000 | 0.0004 | 0.000 | 0.0007 | 0.000** | 0.0003 | 0.000 | -0.0006 | 0.000** | -0.0006 | 0.000*** |
|  |  |  | 0.0004 | 0.000 | 0.0007 | 0.000** | 0.0003 | 0.000 | -0.0006 | 0.000** | -0.0006 | 0.000*** |
| Sex | -0.0232 | 0.012 | 0.0473 | 0.145 | 0.2169 | 0.165 | -0.2295 | 0.123 | 0.1562 | 0.105 | 0.2674 | 0.097** |
|  |  |  | 0.0473 | 0.145 | 0.2169 | 0.165 | -0.2295 | 0.123 | 0.1562 | 0.105 | 0.2674 | 0.097** |
| GCSE | 0.0553 | 0.020** | -0.2743 | 0.264 | -0.5488 | 0.329 | 0.0482 | 0.229 | -0.2279 | 0.187 | -0.3107 | 0.169 |
|  |  |  | -0.2743 | 0.264 | -0.5488 | 0.329 | 0.0482 | 0.229 | -0.2279 | 0.187 | -0.3107 | 0.169 |
| A level | 0.0107 | 0.017 | -0.3531 | 0.200 | -0.3120 | 0.233 | 0.3148 | 0.171 | -0.1058 | 0.147 | 0.1365 | 0.137 |
|  |  |  | -0.3531 | 0.200 | -0.3120 | 0.233 | 0.3148 | 0.171 | -0.1058 | 0.147 | 0.1365 | 0.137 |
| Degree | 0.0095 | 0.016 | -0.0383 | 0.173 | -0.3267 | 0.194 | 0.0548 | 0.151 | -0.1133 | 0.129 | 0.1718 | 0.121 |
|  |  |  | -0.0383 | 0.173 | -0.3267 | 0.194 | 0.0548 | 0.151 | -0.1133 | 0.129 | 0.1718 | 0.121 |
| Sick 1 | -0.1493 | 0.031*** | -0.2101 | 0.265 | 0.2686 | 0.292 | 0.5243 | 0.239* | 0.6324 | 0.209** | 0.7318 | 0.206*** |
|  |  |  | -0.2101 | 0.265 | 0.2686 | 0.292 | 0.5243 | 0.239* | 0.6324 | 0.209** | 0.7318 | 0.206*** |
| Sick 2 | -0.1623 | 0.027*** | 0.2257 | 0.261 | 0.5627 | 0.244* | 0.3060 | 0.225 | 0.4125 | 0.191* | 0.6457 | 0.186** |
|  |  |  | 0.2257 | 0.261 | 0.5627 | 0.244* | 0.3060 | 0.225 | 0.4125 | 0.191* | 0.6457 | 0.186** |
| Sick 3 | -0.1536 | 0.020*** | -0.1462 | 0.192 | -0.0154 | 0.203 | 0.8927 | 0.166*** | 0.4385 | 0.147** | 0.5064 | 0.143*** |
|  |  |  | -0.1462 | 0.192 | -0.0154 | 0.203 | 0.8927 | 0.166*** | 0.4385 | 0.147** | 0.5064 | 0.143*** |
| A little deprived | -0.0047 | 0.017 | 0.0270 | 0.225 | 0.3097 | 0.293 | -0.3715 | 0.200 | 0.0772 | 0.163 | 0.1815 | 0.147 |
|  |  |  | 0.0270 | 0.225 | 2.2291 | 0.748** | -0.3715 | 0.200 | 0.0772 | 0.163 | 0.1815 | 0.147 |
| Very deprived | -0.0207 | 0.016 | -0.0781 | 0.186 | 0.4047 | 0.215 | -0.0489 | 0.157 | 0.1089 | 0.135 | 0.0133 | 0.125 |
|  |  |  | -0.0781 | 0.186 | 0.4047 | 0.215 | -0.0489 | 0.157 | 0.1089 | 0.135 | 0.0133 | 0.125 |
| Most deprived | -0.0625 | 0.017*** | 0.2888 | 0.186 | 0.3963 | 0.208 | -0.2411 | 0.159 | -0.0145 | 0.152 | 0.0510 | 0.161 |
|  |  |  | 0.2888 | 0.186 | 0.3963 | 0.208 | -0.2411 | 0.159 | 0.4636 | 0.196* | 0.4338 | 0.143** |
| GHVG | 0.1865 | 0.018*** | -0.6169 | 0.401 | 0.5840 | 0.461 | -0.9725 | 0.341** | -0.6568 | 0.253** | -1.3454 | 0.203*** |
|  |  |  | -0.6169 | 0.401 | 0.5840 | 0.461 | -0.9725 | 0.341** | -0.6568 | 0.253** | -1.3454 | 0.203*** |
| GHG | 0.1020 | 0.014*** | -0.3604 | 0.187 | -0.2747 | 0.279 | -0.6301 | 0.160*** | -0.1750 | 0.132 | -0.5834 | 0.122*** |
|  |  |  | -0.3604 | 0.187 | -0.2747 | 0.279 | -0.6301 | 0.160*** | -0.1750 | 0.132 | -0.5834 | 0.122*** |
| GHB | -0.2245 | 0.020*** | 0.3810 | 0.182* | 0.3495 | 0.194 | 0.8249 | 0.165*** | 0.2787 | 0.145 | 0.7177 | 0.140*** |
|  |  |  | 0.3810 | 0.182* | 0.3495 | 0.194 | 0.8249 | 0.165*** | 0.2787 | 0.145 | 0.7177 | 0.140*** |
| GHVB | -0.3951 | 0.033*** | 0.2664 | 0.317 | 0.2942 | 0.275 | 1.3552 | 0.267*** | 1.0675 | 0.227*** | -0.0238 | 0.349 |
|  |  |  | 0.2664 | 0.317 | 0.2942 | 0.275 | 1.3552 | 0.267*** | 1.0675 | 0.227*** | 1.1721 | 0.233*** |
| Year2004 | -0.0025 | 0.023 | -0.0246 | 0.257 | -0.0198 | 0.300 | -0.1452 | 0.223 | 0.2206 | 0.189 | -0.1024 | 0.177 |
|  |  |  | -0.0246 | 0.257 | -0.0198 | 0.300 | -0.1452 | 0.223 | 0.2206 | 0.189 | -0.1024 | 0.177 |
| Year2005 | 0.0287 | 0.018 | -0.1620 | 0.223 | -0.1579 | 0.259 | -0.0833 | 0.191 | 0.4178 | 0.177* | -0.2142 | 0.150 |
|  |  |  | -0.1620 | 0.223 | -0.1579 | 0.259 | -0.0833 | 0.191 | -0.2235 | 0.278 | -0.2142 | 0.150 |
| Year2006 | -0.0079 | 0.018 | -0.1006 | 0.205 | -0.0021 | 0.230 | 0.3890 | 0.184* | 0.0206 | 0.149 | -0.0048 | 0.136 |
|  |  |  | -0.1006 | 0.205 | -0.0021 | 0.230 | -0.5331 | 0.328 | 0.0206 | 0.149 | -0.0048 | 0.136 |
| Year2008 | 0.0014 | 0.018 | 0.2583 | 0.198 | -0.0479 | 0.223 | -0.4229 | 0.170* | 0.3320 | 0.144* | -0.1361 | 0.132 |
|  |  |  | 0.2583 | 0.198 | -0.0479 | 0.223 | -0.4229 | 0.170* | 0.3320 | 0.144* | -0.1361 | 0.132 |
| Mobility |  |  |  |  | 1.4056 | 0.210*** | 1.7150 | 0.160*** | 1.8651 | 0.144*** | -0.0161 | 0.136 |
|  |  |  |  |  | 1.4056 | 0.210*** | 0.6038 | 0.361 | 1.8651 | 0.144*** | -0.0161 | 0.136 |
| Self care |  |  | 1.3599 | 0.218*** |  |  | 2.5578 | 0.325*** | 0.5658 | 0.153*** | -0.6525 | 0.212** |
|  |  |  | 1.3599 | 0.218*** |  |  | 1.1574 | 0.250*** | 0.5658 | 0.153*** | 0.0103 | 0.159 |
| Usual act |  |  | 1.6006 | 0.150*** | 1.7596 | 0.190*** |  |  | 0.7023 | 0.118*** | 0.8401 | 0.117*** |
|  |  |  | 1.6006 | 0.150*** | 1.7596 | 0.190*** |  |  | 0.7023 | 0.118*** | 0.8401 | 0.117*** |
| Pain |  |  | 1.6498 | 0.134*** | 0.5658 | 0.144*** | 0.7451 | 0.114*** |  |  | 0.4046 | 0.123** |
|  |  |  | 1.6498 | 0.134*** | 0.5658 | 0.144*** | 0.7451 | 0.114*** |  |  | -0.0484 | 0.110 |
| Anxiety |  |  | 0.0397 | 0.118 | -0.1118 | 0.133 | 0.7263 | 0.102*** | 0.1690 | 0.085* |  |  |
|  |  |  | 0.0397 | 0.118 | -0.1118 | 0.133 | 0.7263 | 0.102*** | 0.1690 | 0.085* |  |  |
| Constant | 0.7773 | 0.050*** | -8.2353 | 0.788*** | -6.7791 | 0.856*** | -7.0869 | 0.713*** | -6.5175 | 0.560*** | -0.5423 | 0.505 |
|  |  |  | -18.2037 | 1.388*** | -11.5903 | 0.979*** | -9.6511 | 0.970*** | -10.2420 | 0.608*** | -3.8042 | 0.485*** |

^a^As the PPOMs comprise of two equations, each explanatory variable has two rows with two Beta coefficients (and corresponding SE). *p<0.05; **p<0.01; ***p<0.001
